# An in vitro study on the efficacy of nanoparticles and nanocomposites as coating materials on surgical sutures

**DOI:** 10.1038/s41598-025-07558-6

**Published:** 2025-07-01

**Authors:** Heba R. Shebl, Rehab A. Soliman, Omnia M. Abdallah

**Affiliations:** 1 Microbiology Department, Faculty of Dentistry, Misr International University, Cairo, Egypt; 2 Oral and Maxillofacial Surgery Department, Faculty of Dentistry, Misr International University, Cairo, Egypt

**Keywords:** Coated sutures, Silver nanoparticles, Nanocomposite, Atomic force microscopy, Field emission scanning electron microscopy, Antibacterial, Biological techniques, Biotechnology, Microbiology, Nanoscience and technology

## Abstract

Upgrading the surgical sutures, as the main wound closure device, is essential. The evolution of bacterial resistance and the plummeting of antibiotics have directed research toward augmented sutures. Nanotechnology has provided answers to these concerns. The use of bacterial isolates as bio-factory for synthesized silver nanoparticles (AgNPs) and silver nanocomposites via a one pot ex situ method provides environmentally friendly silver nanocomposites in addition to the use of chitosan and polyvinyl alcohol polymers as carriers. Transmission electron microscopy (TEM) and zeta potential analysis revealed spherical negatively charged AgNPs. These nanoparticles and nanocomposites were used as coatings for absorbable vicryl and nonabsorbable silk surgical sutures. Atomic force microscopy (AFM) 3D images of these coated sutures showed a significant decrease in surface roughness with improved surface topography, specifically with chitosan-silver (CS-Ag) vicryl coated sutures with effective attachment of the nanocomposite and nanoparticles thin film on the suture surface. Field emission scanning electron microscopy with energy dispersive x-ray spectroscopy (FE-SEM/EDX) analysis showed the significant presence of the thin film of coating materials on the surface of the sutures and the significant elemental presentation of Ag. Vicryl and silk coated CS-Ag sutures showed significant antibacterial and antibiofilm activities against both gram positive and gram-negative bacterial isolates. AgNPs coated silk and vicryl sutures recorded the lowest amounts of Ag ions at 0.03–0.45 ppm released after 14 days, while polyvinyl alcohol-silver (PVA-Ag) coated ones showed the highest rates at 0.75–0.93 ppm.

**Supplementary Information:**

The online version contains supplementary material available at 10.1038/s41598-025-07558-6.

## Introduction

Surgical sutures are among the most important surgical devices used for wound closure. This type of filament-shaped device is one of the oldest and most common surgical device and cannot be outdated by modern technology in the medical field^1^. However, it can be augmented with other properties to increase its efficiency and limit its drawbacks. The main function of sutures is closure of wounds and incision sites, and they can be classified according to their absorbability and biodegradability. They are made of dissimilar materials with variable absorbability ranges such as silk, nylon, cut gut and vicryl sutures^2^. One of the major suture complications is the postsurgical infection (PSI) or site surgical infection (SSI) because bacterial growth and biofilm formation cause postoperative tissue inflammation surrounding the surgical site^3,4^. Sutures serve as a niche for bacterial attachment, proliferation and eventually biofilm formation. This major drawback significantly increases the infection risk by 10,000 times. SSI increases mortality rates and is considered the most common hospital acquired infection. Furthermore, it strongly impacts the number of days of hospitalization and increases the cost of medical services per patient^5,6^. In 2011, the WHO reported that SSI was estimated to affect more than 20% of patients as postsurgical complication, increasing the use of systemic and localized antibiotics, which are associated with rouge and drug-resistant bacteria^7^. This leads to a vicious cycle of repeated drug-resistant infections and the extreme use of antibiotics. Surgical sutures can be sterilized by autoclaving or chemical disinfection, nevertheless, these actions cannot guarantee the prevention of microbial attachment and growth once the sutures are used at surgical sites^8^. Bacterial growth and biofilm formation are complex processes at surgical sites. Both processes start with bacterial adhesion to suture materials after the implantation procedure and, consequently, proliferation and infection. Therefore, recent studies have focused on the initial prevention of this adhesion step and hence preventing the formation of biofilms with virulent and multi- drug resistant (MDR) bacteria^1,9^. Recently, coating surgical sutures with antibiotics has been adopted to add antimicrobial activity to sutures by limiting their susceptibility to bacterial growth and lowering surgical site complications. Multiple sutures have been improved by different antibiotics such as ciprofloxacin, levofloxacin hydrochloride, octenidine and chlorhexidine to reduce bacterial growth. However, this step faces the emergence and spread of MDR bacteria, causing an abbreviated period of bacterial inhibition and increasing the risk of wound healing and inflammatory reactions^10,11^. Therefore, the demand for an effective alternative was proposed. Currently, the direct delivery of antimicrobial products from sutures to scared tissue is used and targeted by many studies. Nanoparticle (NP) and nanocomposite (NC) coatings are the most developed alternatives to tackle microbial growth associated with surgical sites^12^. Coating surgical sutures has been reported as an effective strategy to increase their ability to fight microbial growth and prevent the formation of biofilms. Various physical, chemical, and biological pathways can be used to synthesize NPs. Biologically synthesized NPs can be achieved via the use of bacterial isolates as bio factories for the synthesis of stabilized NPs, where the bacterial cell structure provides an effective metabolic pathway for the bio-formation of NPs with well-defined shapes, high reactivity, and water solubility properties ^9,13^. These findings suggested that biologically synthesized NPs are the favored choice for many biomedical applications^13^. Silver nanoparticles (AgNPs) are among the most promising antimicrobial agents with a wide range of applications, such as in wound and burn healing, as well as in bone and dental implants^14^. These nanoparticles can be bound to polymers that form NCs with antimicrobial properties. Polyvinyl alcohol (PVA) and chitosan (CS) have been integrated into many applications because they are biodegradable and biocompatible. PVA is FDA approved for use in the food industry and medical applications without side effects. Furthermore, CS is a natural polymer that can be hydrolyzed by a human enzymatic system into harmless products. Both PVA and CS can be used as biological scaffolds for nanoparticles, leading to a wider scope of applications^13,15^. In our study, biologically synthesized AgNPs were used, and the sol–gel coating method was employed to coat nonabsorbable silk and absorbable vicryl surgical sutures. Coated sutures were evaluated for their antimicrobial and antibiofilm activities against various bacterial isolates.

## Materials and methods

### Biosynthesis of silver nanoparticles and nanocomposites

**Fig. 1:**
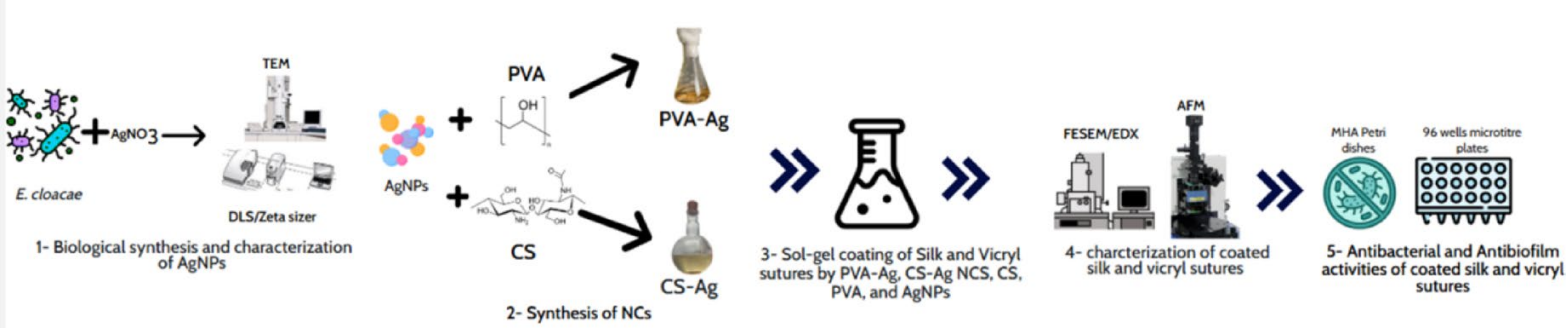
A schematic diagram of experimental setup.

A schematic diagram of experimental setup is shown on Fig. 1. Locally isolated *Enterobacter cloacae* Ism 26 (KP988024) was used to biosynthesis AgNPs. Briefly, a bacterial culture was inoculated in 100 mL of nutrient broth medium, incubated at 35 °C for 24 h, and then centrifuged for 15 min. Bacterial supernatant was mixed with AgNO_3_ (1 mM) and incubated at 35 °C for 24 h. AgNPs synthesis will be indicated visually by forming brown color. Synthesized AgNPs was lyophilized via an Edwards model RV5 (England). Biosynthesized AgNPs were used at six different concentrations (C) (W/V%) (0.1% (C1), 0.2% (C2), 0.3% (C3), 0.4% (C4), 0.5% (C5) and 0.6% (C6)) in further experiments. The synthesized AgNPs were mixed with PVA or CS separately to form silver nanocomposites. For synthesis of PVA-Ag nanocomposite, 2 g of PVA (Alpha Cheimeka, India) was added to 20 ml of deionized water and magnetically stirred on a hot plate at 90 °C for 3 h. Then, different concentrations of the biosynthesized AgNPs were added and stirred for another 4 h (from C1-C6). For CS-Ag nanocomposite, 0.4 g of CS was added to 20 ml of acetic acid (1%) and magnetically stirred for 1 h at 60 °C; then, different concentrations of AgNPs were added (from C1-C6), and the mixture was further stirred for 2 h. These nanocomposite solutions were used for further experiments. Pure PVA and CS solutions were used as controls^15^.

### Sol–gel coating of surgical sutures with AgNPs and nanocomposites

Vicryl (PGA-absorbable braided 4-0, Assucryl sutures, Switzerland) and silk (nonabsorbable braided 3-0, Assut medical sutures, Switzerland) sutures were immersed for 24 h in different AgNPs, PVA-Ag and CS-Ag concentrations of nanoparticles and silver nanocomposites sol–gel solutions. After 24 h, all samples were grasped with sterilized tweezers and left to air dry in a laminar flow hood overnight. Then, all coated sutures were placed in an oven at 40 °C for 10 min. Beside uncoated sutures, sutures were coated by PVA and CS only and were used as control. All the procedure steps were conducted under laminar flow, and all the samples were separated and sealed in sterilized Eppendorf tubes until the next experiments were performed^13,16^.

### Characterization of silver nanoparticles and coated sutures

Biologically synthesized AgNPs were characterized via UV–Vis spectrometry to detect specific peaks (400–450 nm), dynamic light scattering (DLS) and the zeta potential to determine the particle size and surface charge using a PSS-NICOMP particle sizer 380ZLS (Malvern Instruments Ltd.). Accurate nanoparticle shape and size in nm were identified via transmission electron microscopy** (**TEM) (JOEL JEM-1010) at 80 kV at the Regional Centre for Mycology and Biotechnology (RCMB) of Al-Azhar University^15^. The surface topography and roughness of the uncoated and coated vicryl and silk sutures were determined via atomic force microscopy (AFM) (NanoSurf C3000, Gräubernstrasse, Liestal, Switzerland) operating in phase contrast mode. AFM provides 3D images with measurements of surface roughness, and irregularity in defined measured areas. The average thickness, roughness (Ra), and maximum roughness depth (Rq) were calculated for uncoated and coated sutures using image processing and data analysis software supplied with the AFM^4^. Furthermore, coated, and uncoated sutures were examined by field emission scanning electron microscopy with energy dispersive X-ray spectroscopy (FE-SEM/EDX) (QUANTA, FEG 250, Thermo Scientific) operating at an accelerating voltage of 30 kV to visualize the surface changes and detect the extent of the coating and its efficacy on the suture surface for all the tested coating materials vs. the uncoated control suture samples. EDX analysis was employed to calculate and identify the composition and elemental analysis of each sample surface. The suture samples were mounted on metallic copper stubs and fixed with carbon conductive tape at a standard tilt angle, and FE-SEM photomicrographs were taken from the surface at various magnifications^7,17^. Uncoated sutures served as controls throughout the experiments.

### Antibacterial activity of coated and uncoated sutures

The antibacterial activity of all the coated and uncoated sutures was evaluated using an agar diffusion test according to the Clinical and Laboratory Standards Institute (CLSI). This test was performed against clinically relevant three Gram-positive *Staphylococcus aureus* (S1), *Streptococcus mutans* (St1), and *Enterococcus faecalis* (E1) and three Gram-negative bacterial microorganisms *Acinetobacter baumannii* (A1), *Acinetobacter baumannii* (A2), and *Pseudomonas aeruginosa* (P1). Muller-Hinton agar (MHA) plates were inoculated with 1 × 10^6^ CFU/ml of each bacterial culture separately, six suture samples were placed on each plate (uncoated control, PVA-coated, CS-coated, AgNPs-coated, PVA-Ag coated, and CS-Ag coated). Finally, MHA plates were incubated at 37 °C for 24. The antibacterial activity was evaluated by measuring the diameter of the inhibition zone (mm); the results were reported as the mean ± standard deviation^13,15,16^.

### Antibiofilm activity of coated and uncoated sutures

The ability of coated and uncoated silk and vicryl sutures to inhibit biofilm formation was assessed by using *Acinetobacter baumannii* (A1), and *Pseudomonas aeruginosa* (P1) strong biofilm forming bacterial isolates^13,15^. A1 and P1 biofilm forming bacteria were inoculated, separately, in 20 ml of tryptic soy broth (TSB) (Merck, Germany) supplemented with 1% glucose and incubated at 37 °C for 24 h. Using a 96-well microtiter plate, coated and uncoated sutures were added at the bottom of each well, then 200 μl of each diluted bacterium (1:100) was inoculated into the wells. Finally, these microtiter plates were incubated at 37 °C for 24 h. After incubation, each well was washed using phosphate buffered saline (pH 7.2) then left to dry for 30 min at room temperature. The next step was the addition of crystal violet (CV) solution (0.1% w/v), next plates were washed using the same buffer solution and left to dry. By the end of these steps, 100 μl of ethanol (96%) was added to each well to extract the stained bound biofilm, and the CV absorbance optical density (OD) at 490 nm with a microplate Reader (ELx808™ Absorbance, Biotek, USA) was measured and graphed. Biofilm inhibition was measured, and the percentage of inhibition was calculated using the following equation:

$$\% \;{\text{inhibition}} = 1{-}\left( {{\text{OD}}\;{\text{of}}\;{\text{coated}}\;{\text{sutures}}/{\text{OD}}\;{\text{of}}\;{\text{negative}}\;{\text{control}}} \right) \times 100$$

where the OD of the coated sutures is the optical density of the sample.

The OD of the negative control was the control for biofilm-forming bacteria.

### Quantification of released AgNPs from coated sutures

The amount of AgNPs released from the coated sutures was determined via Atomic Absorption Spectrometry (AAS) (Perkin Elmer 3100) after storage in phosphate-buffered saline for 14 days. The coated sutures were digested in nitric acid, and the concentration of AgNPs released was quantified.

### Statistical analysis

The SPSS standard software package was used for data analysis. One-way analysis of variance (ANOVA) with Tukey’s post- hoc test was used to compare the effects between groups (n = 5). The data are presented as the means ± standard deviation (SDs). The level of significant difference was set at p < 0.05.

## Results

### Characterization of the nanoparticles and coated sutures

**Fig. 2:**
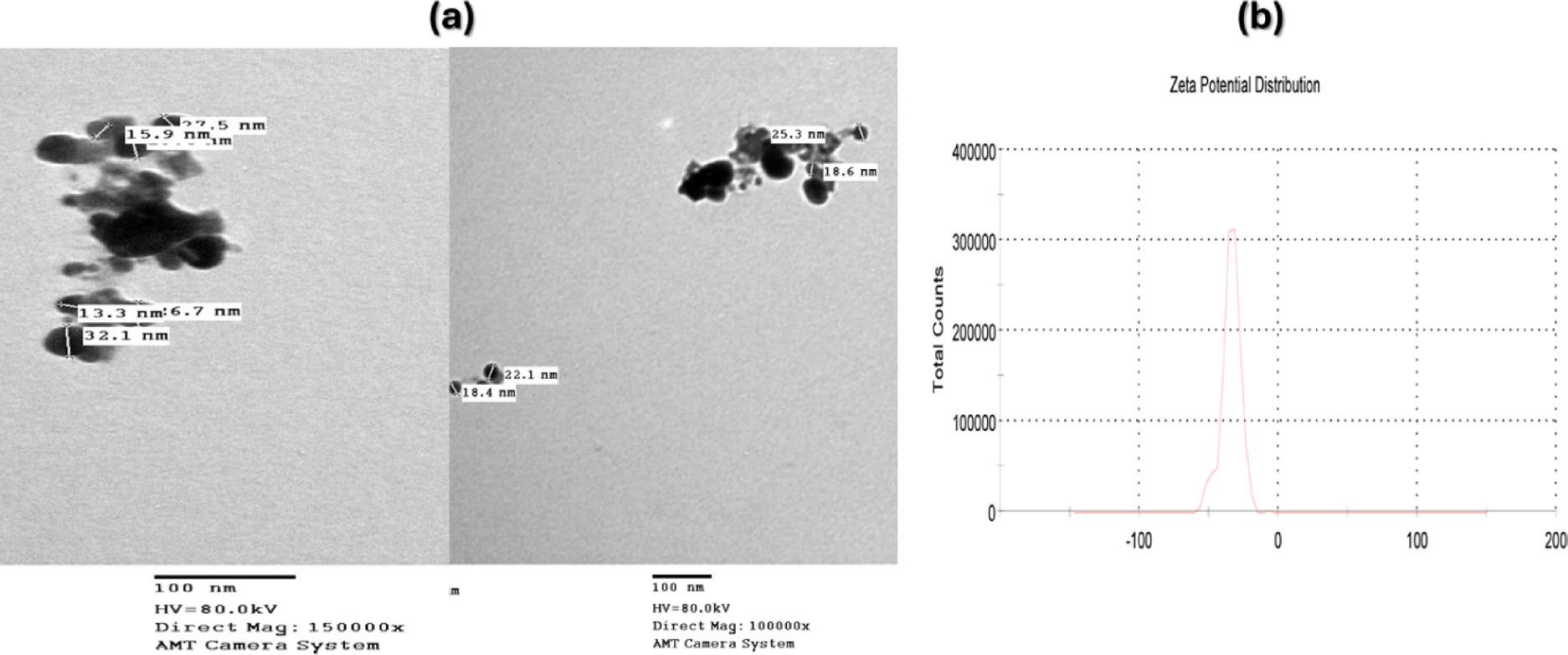
(**a**) TEM micrographs of AgNPs and (**b**) Zeta potential of biologically synthesized AgNPs.

AgNPs synthesized by *Enterobacter cloacae* Ism 26 (KP988024) were characterized, Fig. 2 shows the shape, size and electric charge of synthesized nanoparticles surface. TEM micrographs in Fig. 2a showed rounded to spherical shaped nanoparticle sizes ranging from 33 to 14 nm, with an average size of 15 nm. Figure 2b showed negatively charged nanoparticles with a zeta potential of -34 mV. Using the sol–gel coating technique, silk and vicryl sutures were immersed in various concentrations of AgNPs and nanocomposites sol–gel solutions.

**Fig. 3:**
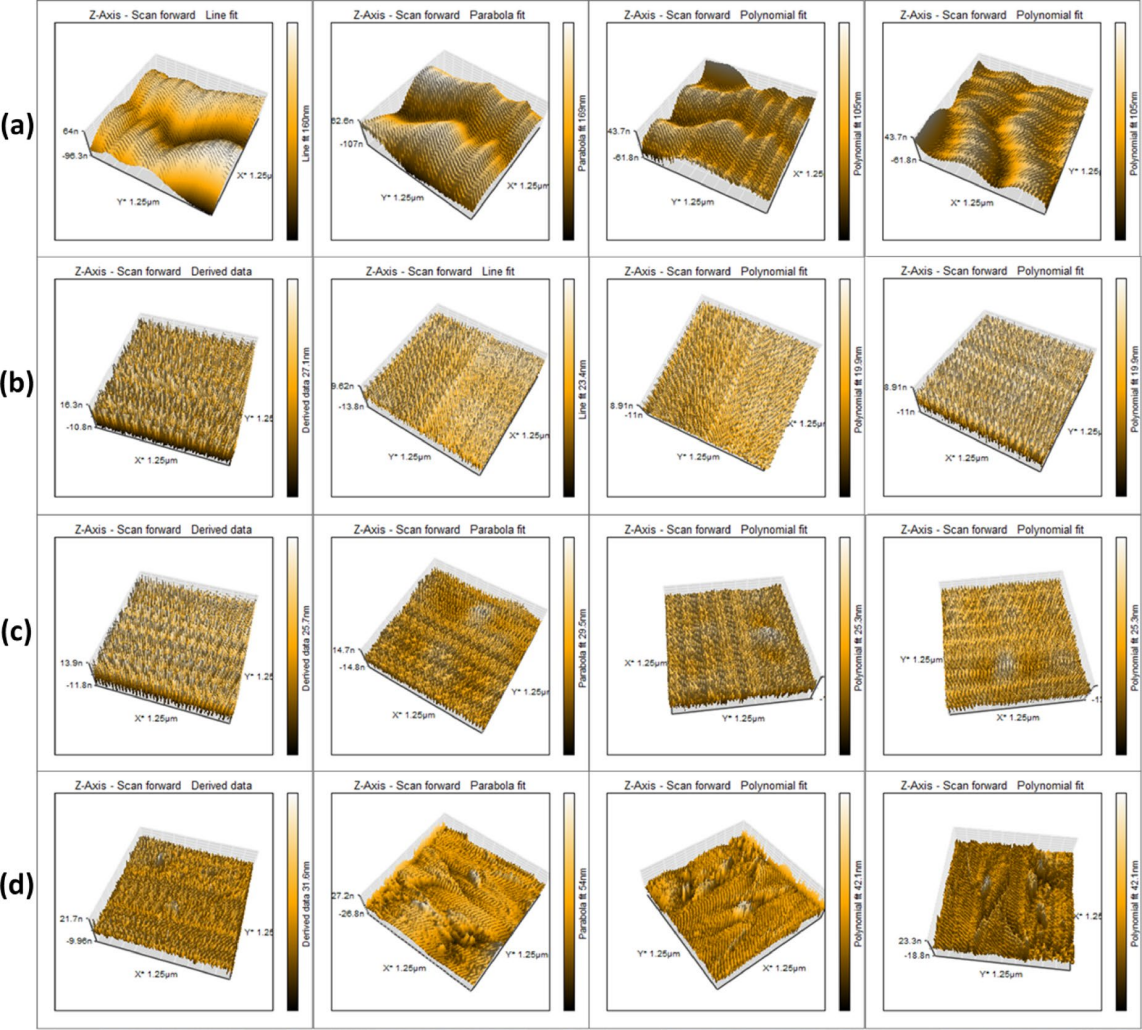
AFM 3D pictures of vicryl sutures. (**a**) control uncoated vicryl suture, (**b**) CS-Ag coated, (**c**) PVA-Ag coated and (**d**) AgNPs coated vicryl sutures.

**Fig. 4:**
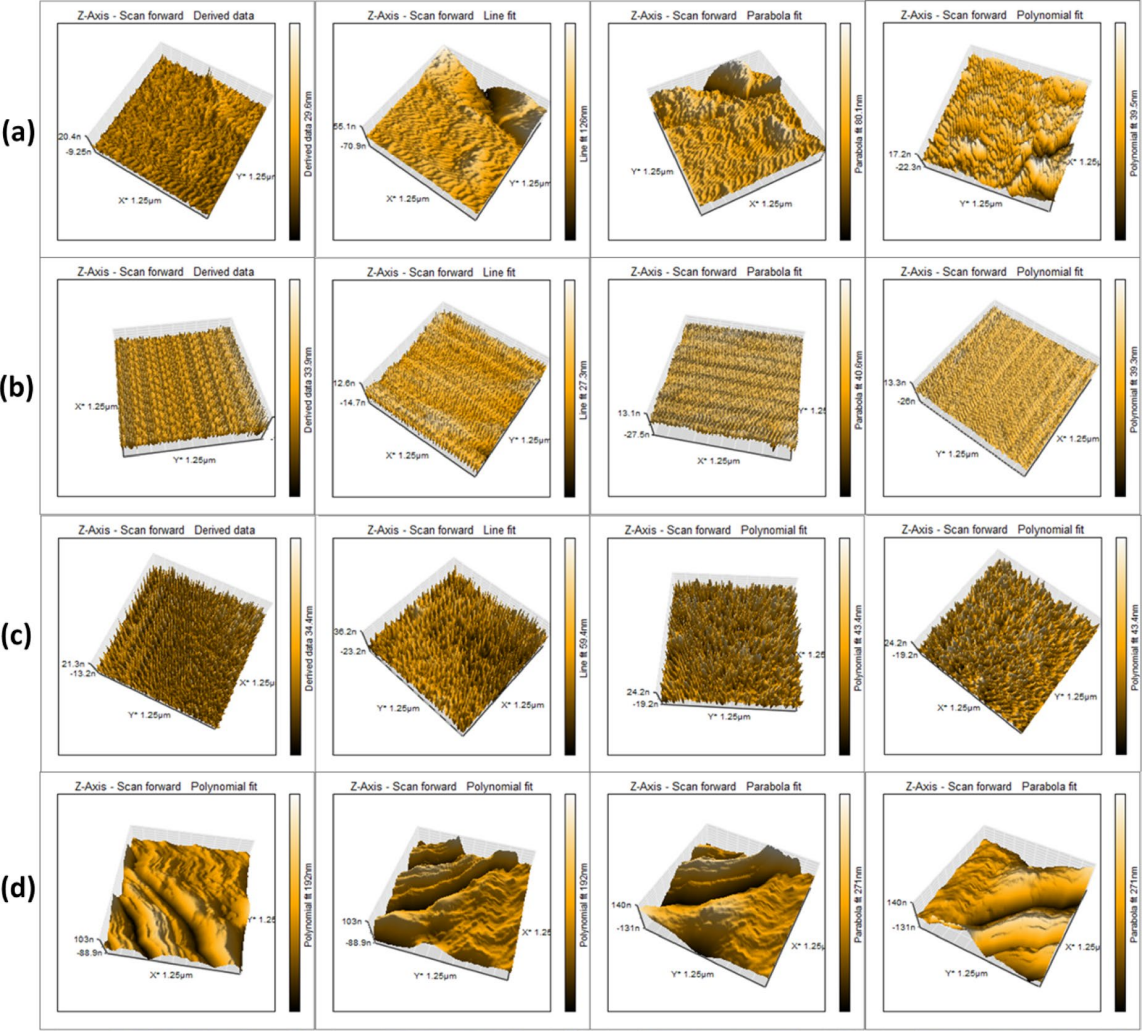
AFM 3D pictures of silk sutures. (**a**) Control uncoated silk suture, (**b**) CS-Ag coated, (**c**) PVA-Ag coated and (**d**) AgNPs coated silk sutures.

AFM was performed for coated and uncoated sutures via different fitting techniques to model the coating data and results. 3D image pseudo-colored graphs are shown in Figs. 3 and 4, which reveal that the thickness and surface roughness changed according to the different coating materials. The variability in thickness varied from silk to vicryl sutures and from one coating material to another. For the vicryl sutures, using polynomial fit, all coated sutures varied in thickness, with average thickness of 19.7, 25.7, and 42.1 nm, for the CS-Ag, PVA-Ag, and AgNPs samples, respectively, while uncoated suture showed thickness of 105 nm. Vicryl sutures with different coating materials showed variable thickness. Furthermore, by measuring the average Ra values, the data revealed a significant decrease in roughness, where uncoated vicryl sutures had an Ra value of 12.6 nm, and coated sutures had Ra values of 4.71, 7.09 and 2.80 nm for CS-Ag, PVA-Ag and AgNPs, respectively. Rq values were also measured showing variable values according to the coating material, where uncoated vicryl sutures had an Rq value of 14.13 nm, and coated sutures had much lower values of 5.37, 8.11 and 3.67 nm for CS-Ag, PVA-Ag and AgNPs, respectively. Uncoated and coated silk sutures were also measured and results were recorded. Using polynomial fit, the thickness of coated silk sutures varied widely on average at 39.2, 43.4 and 192 nm for CS-Ag, PVA-Ag, and AgNPs, respectively, where uncoated silk suture showed thickness of 39.5. By measuring the average Ra values, the data showed variation according to the type of coating material, where the control uncoated silk sutures had an Ra value of 2.3 nm, and the CS-Ag coating had an average value of 1.87 nm. However, the PVA-Ag coating increased the Ra value to 5.7 nm, whereas the AgNPs coating significantly increased Ra value to 124.03 nm. The Rq values were also recorded with 3.01 nm for the uncoated silk sutures, a slightly lower value for CS-Ag at 2.20 nm and an increased value for the PVA-Ag coating. The AgNPs coating showed a remarkably high Rq value at 146.7 nm (Supplementary Fig. S1, and S2).

### Field emission scanning electron microscopy (FE-SEM)/energy dispersive x-ray spectroscopy (EDX)

**Fig. 5:**
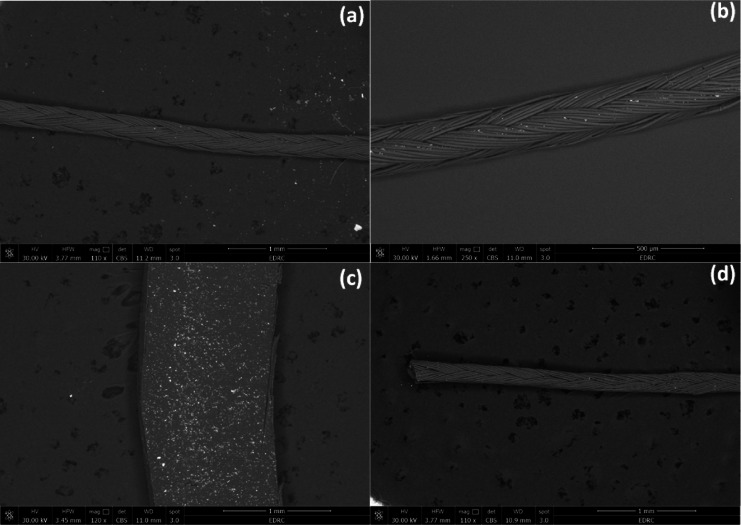
FE-SEM pictures of absorbable vicryl sutures. (**a**) Control uncoated vicryl suture, (**b**) PVA-Ag coated, (**c**) CS-Ag coated and (**d**) AgNPs coated vicryl sutures.

**Fig. 6:**
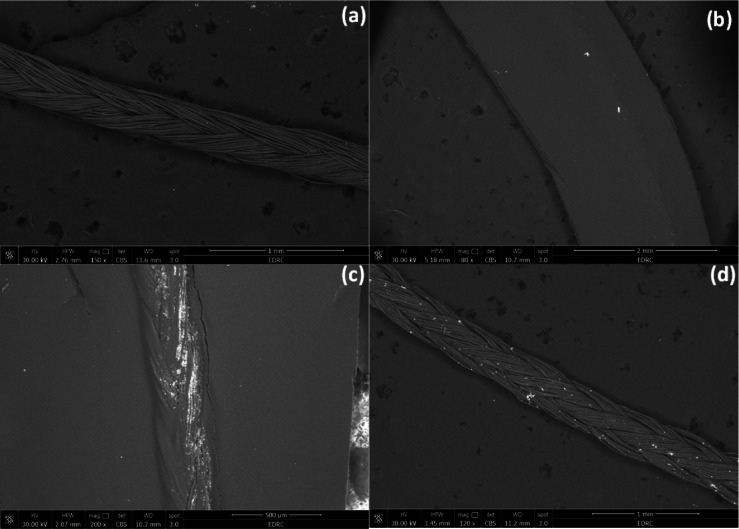
FE-SEM pictures of non-absorbable silk sutures. (**a**) Control uncoated silk suture, (**b**) PVA-Ag coated, (**c**) CS-Ag coated and (**d**) AgNPs coated silk sutures.

**Fig. 7:**
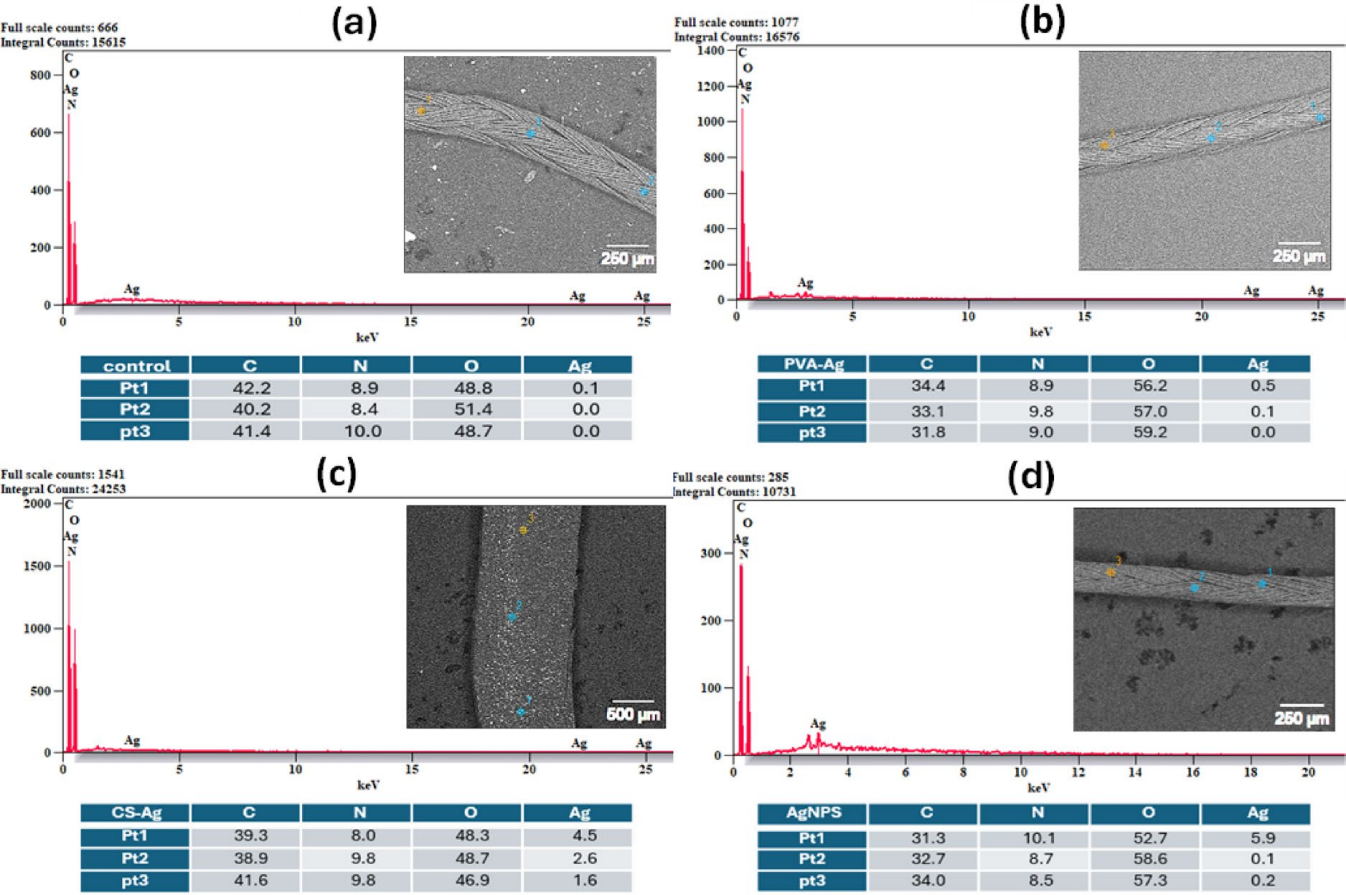
EDX analysis with element percentiles of vicryl sutures. (**a**) Control uncoated vicryl suture, (**b**) PVA-Ag coated, (**c**) CS-Ag coated and (**d**) AgNPs coated vicryl sutures.

**Fig. 8:**
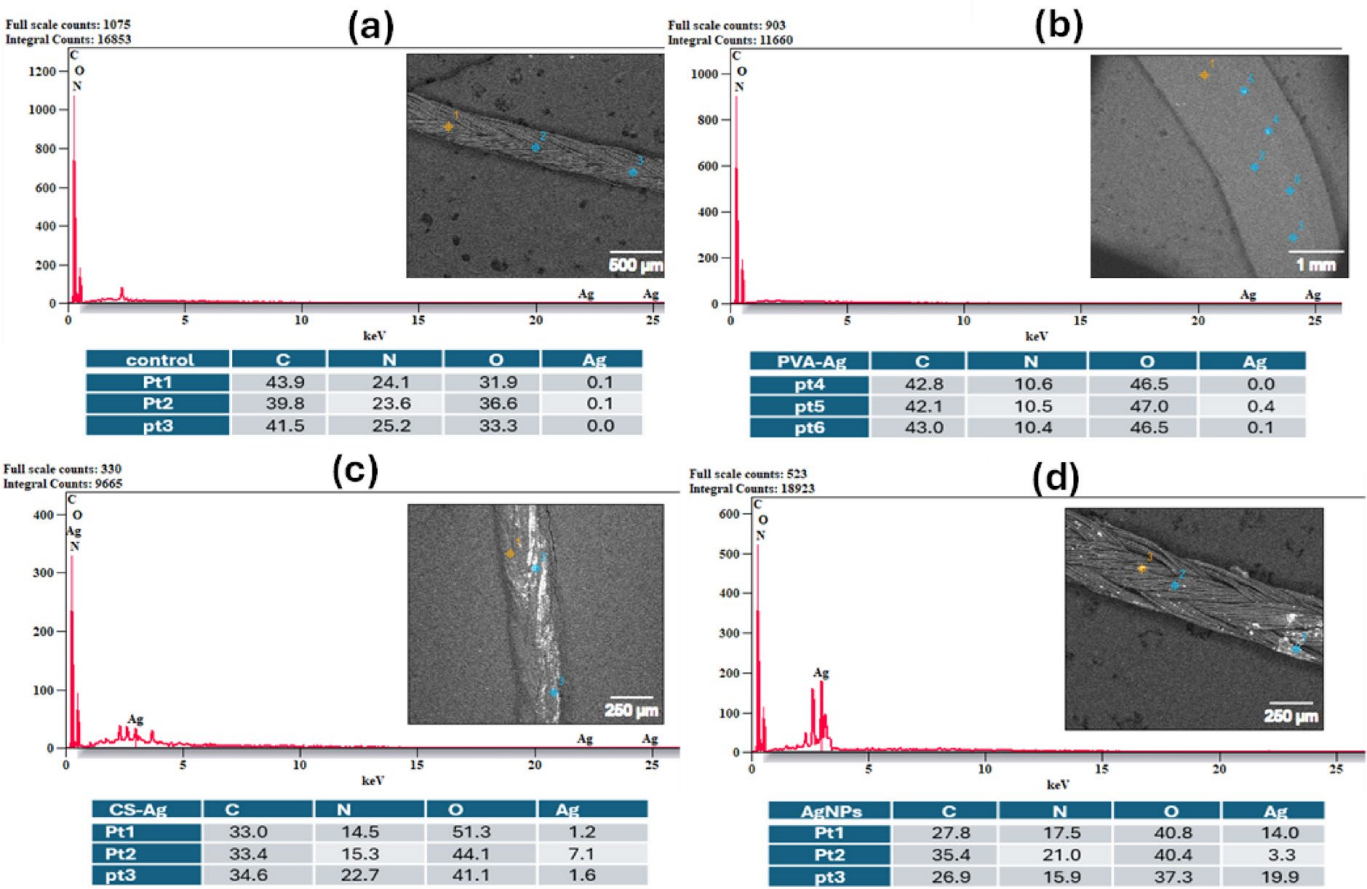
EDX analysis with element percentiles. (**a**) Control uncoated silk suture, (**b**) PVA-Ag coated, (**c**) CS-Ag coated and (**d**) AgNPs coated silk sutures.

The FE-SEM/EDX micrographs of the uncoated and coated silk and vicryl sutures showed the effect of different coating materials on the suture surfaces. FE-SEM images confirmed the coverage of the coating layer on the surface of the silk and vicryl sutures as shown in Figs. 5 and 6. By comparing uncoated silk and vicryl sutures with coated ones, the impact of coating with nanoparticles and nanocomposites was photographed, and all the coated sutures showed complete coverage of the material used on the suture surface by bright spots imbedded within the braided structure of the sutures without any effect on the integrity of the suture braided structure. EDX analysis was used to examine the elemental composition of the coated sutures, which displayed the presence of major elements such as carbon (C), nitrogen (N) and oxygen (O), which are the main components of silk and vicryl sutures in addition to CS and PVA as presented in Figs. 7 and 8. Furthermore, the elemental presence of silver (Ag) was evident in all the coated sutures confirming the presence and attachment of silver ions on the surface of the sutures and absent from all uncoated sutures as expected. Additionally, the presence of Ag was very low and sometime undetected with PVA-Ag as a coating material for both silk and vicryl sutures. However, Ag presence was more significant with the CS-Ag coating layer with silk and vicryl sutures with number of bright spots indicating the presence and attachment of Ag. For the AgNPs as a coating layer showed high amounts of Ag atoms for both types of sutures.

### Antibacterial activity of coated and uncoated sutures

**Fig. 9:**
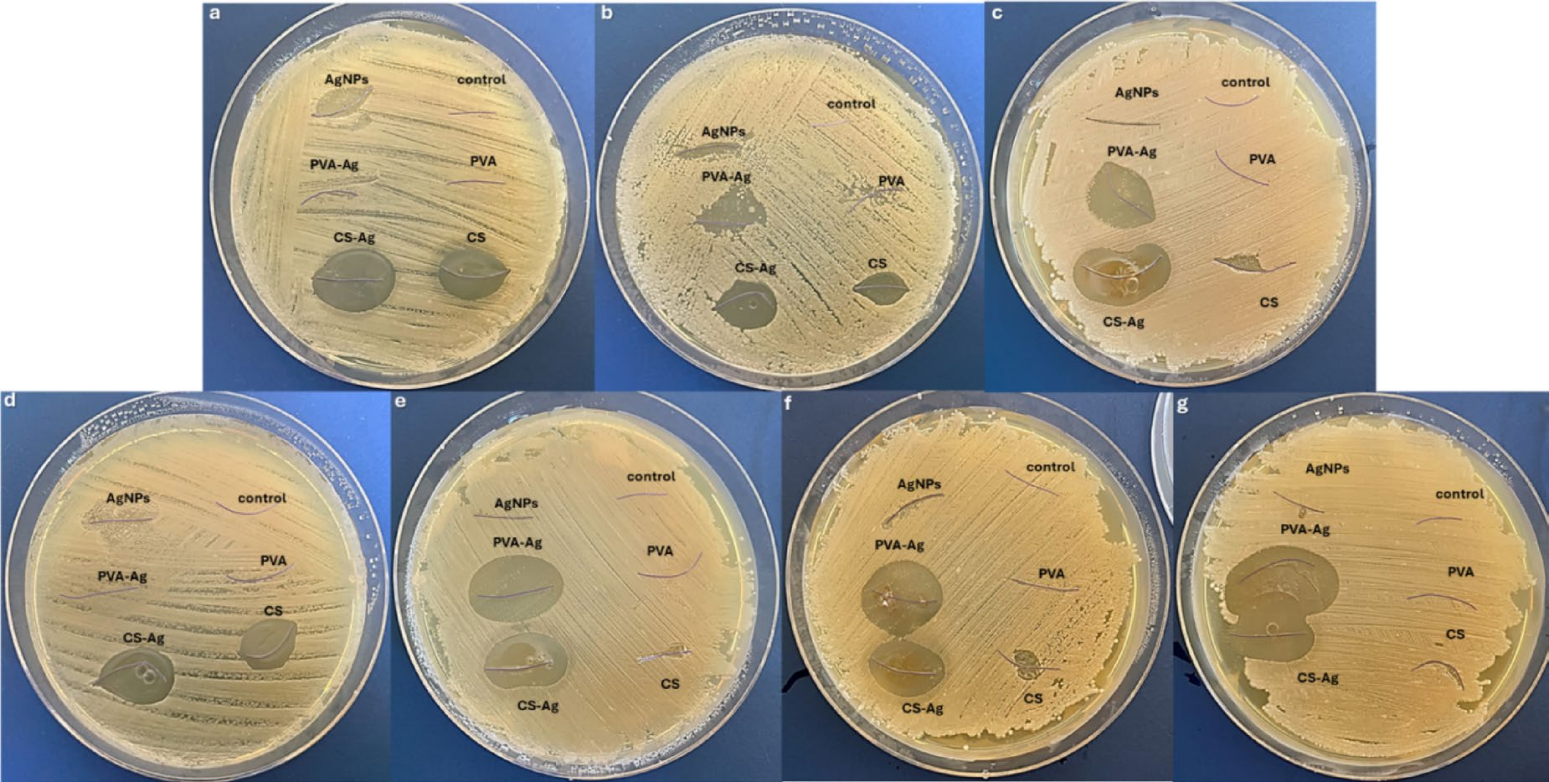
Antibacterial activity of coated and uncoated absorbable vicryl sutures at ascending concentrations of AgNPs, CS-Ag and PVA-Ag. (**a**–**g**) Muller-Hinton agar plates cultured with Gram positive *Staphylococcus aureus* (S1), *Streptococcus mutans* (St1), and *Enterococcus faecalis* (E1) and Gram-negative *Acinetobacter baumannii* (A1), *Acinetobacter baumannii* (A2), and *Pseudomonas aeruginosa* (P1) bacterial isolates.

**Fig. 10:**
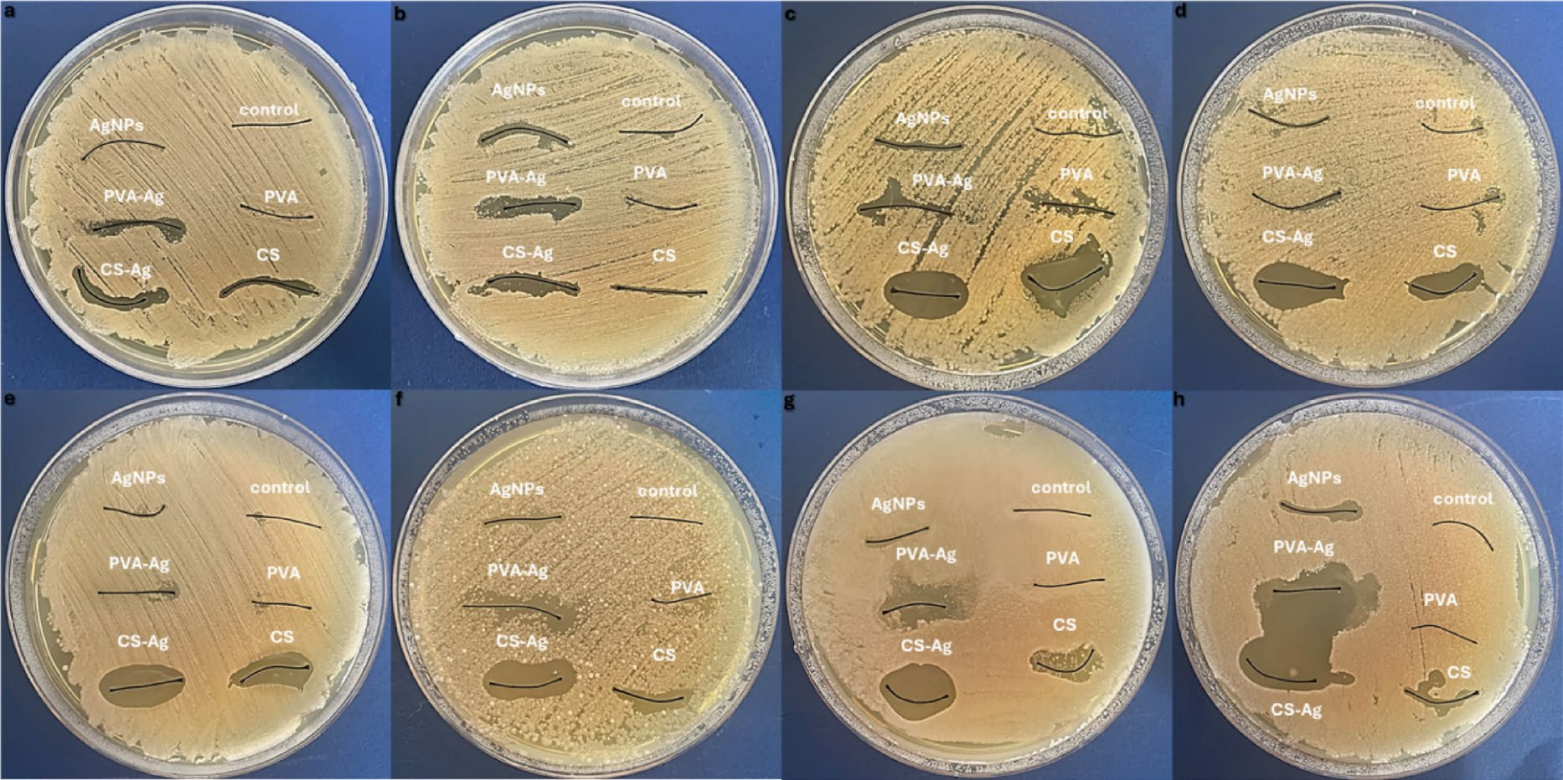
Antibacterial activity of coated and uncoated unabsorbable silk sutures at ascending concentrations of AgNPs, CS-Ag and PVA-Ag. (**a**–**h**) Muller hinton agar plates cultured with Gram positive *Staphylococcus aureus* (S1), *Streptococcus mutans* (St1), and *Enterococcus faecalis* (E1) and Gram-negative *Acinetobacter baumannii* (A1), *Acinetobacter baumannii* (A2), and *Pseudomonas aeruginosa* (P1) bacterial isolates.

**Fig. 11:**
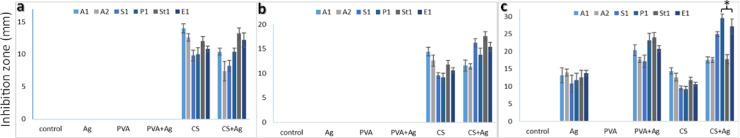
Ggraphical representation of antibacterial activity with inhibition zone. (**a**–**c**) (C1, C2, and C3) ascending concentrations of AgNPs, CS-Ag and PVA-Ag coated and uncoated vicryl sutures against Gram-positive *Staphylococcus aureus* (S1), *Streptococcus mutans* (St1), and *Enterococcus faecalis* (E1) and Gram-negative bacterial microorganisms *Acinetobacter baumannii* (A1), *Acinetobacter baumannii* (A2), and *Pseudomonas aeruginosa* (P1).

The antibacterial activities of the coated and uncoated silk and vicryl sutures were evaluated at different concentrations of AgNPs and nanocomposites as coating layers. The antimicrobial effect of AgNPs and Ag nanocomposite vicryl-coated sutures was evident at different concentrations causing range of inhibition zones against all tested Gram positive and Gram-negative bacteria as shown in Fig. 9. However, the uncoated vicryl sutures and PVA only coated sutures showed no antibacterial effects at all concentrations. While, adding AgNPs to PVA forming PVA-Ag nanocomposite as a coating layer on vicryl sutures showed antimicrobial activity at 0.3% (w/v), as lower concentrations did not show significant bacterial inhibition. On the other hand, using CS and CS-Ag as a coating material on both silk and vicryl sutures showed significant inhibitory effect on all bacterial isolates, and the CS-Ag coated sutures exhibited significant antimicrobial activity that was directly proportional to the AgNPs concentration as shown in Fig. 10. The inhibition zones varied from one bacterial isolate to another showing the following results: Gram positive *Staphylococcus aureus* (S1) 25 ± 0.70 mm; *Streptococcus mutans* (St1), 17.8 ± 1.30 mm; and *Enterococcus faecalis* (E1), 27.2 ± 2.16 mm; and Gram-negative bacterial isolates *Acinetobacter baumannii* (A1), 17.6 ± 0.89 mm; *Acinetobacter baumannii* (A2), 17.8 ± 0.44 mm; and *Pseudomonas aeruginosa* (P1), 29.6 ± 1.10 mm, as shown in Fig. 11.

**Fig. 12:**
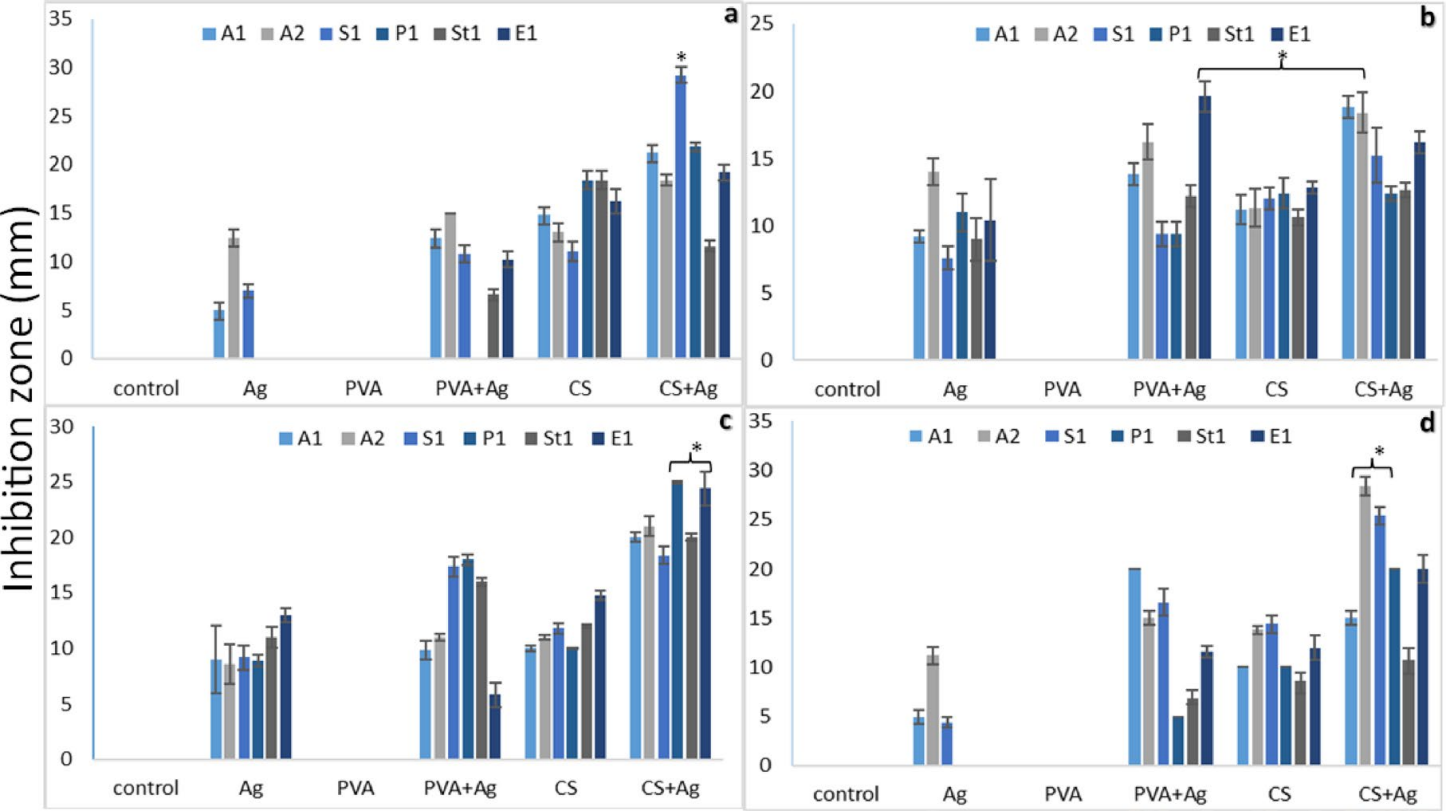
Ggraphical representation of antibacterial activity with inhibition zone. (**a**–**d**) (C3, C4, C5 and C6) ascending concentrations of AgNPs, CS-Ag and PVA-Ag coated and uncoated vicryl sutures against Gram-positive *Staphylococcus aureus* (S1), *Streptococcus mutans* (St1), and *Enterococcus faecalis* (E1) and Gram-negative bacterial microorganisms *Acinetobacter baumannii* (A1), *Acinetobacter baumannii* (A2), and *Pseudomonas aeruginosa* (P1).

Silk sutures, both coated and uncoated, showed no antimicrobial activity at 0.1% (w/v) or 0.2% (w/v). However, as the AgNPs and nanocomposites concentrations increased, antimicrobial activity was observed. At 0.3% (w/v), the number of bacterial species affected by the concentration increase was noteworthy. The most significant concentrations of AgNPs and nanocomposite as a coating layer on silk sutures were 0.5% (w/v) and 0.6% (w/v) and the inhibition effect reached a steady value with intersecting inhibition zones on the MHA plates. CS-Ag coated silk at 0.6% (w/v) showed the most significant antimicrobial activity against all the gram-positive *Staphylococcus aureus* (S1) 25.4 ± 0.9 mm; *Streptococcus mutans* (St1) 10.8 ± 1 mm; and *Enterococcus faecalis* (E1) 20 ± 1.4 mm; and the gram-negative bacterial isolates *Acinetobacter baumannii* (A1) 15 ± 0.7 mm; *Acinetobacter baumannii* (A2) 28.4 ± 0.8 mm, and *Pseudomonas aeruginosa* (P1) 20 mm in size, as shown in Fig. 12.

### Antibiofilm activity of coated and uncoated sutures

**Fig. 13:**
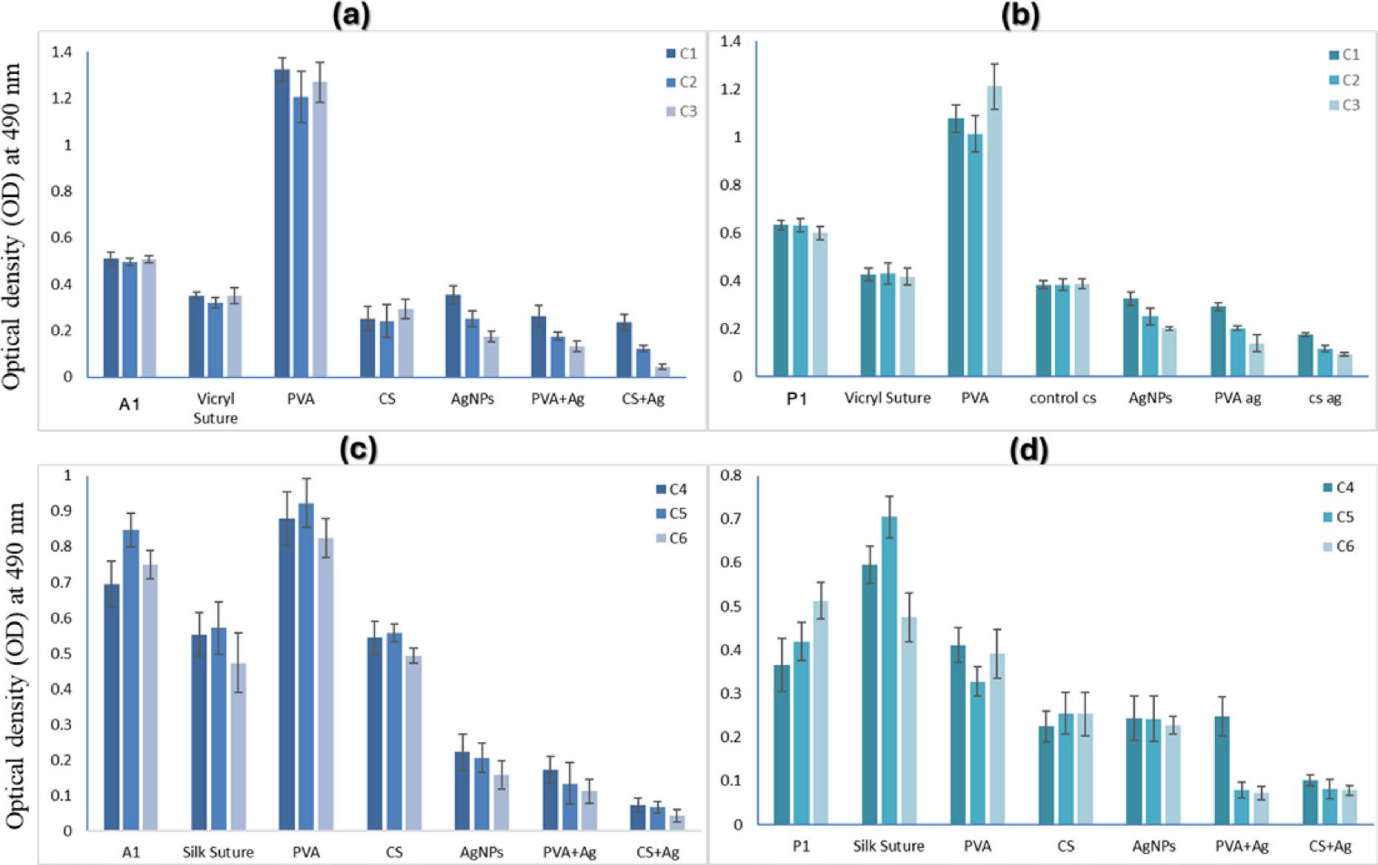
Antibiofilm activity of the uncoated and the coated sutures. (**a**, **b**) vicryl sutures, (**c**, **d**) silk sutures against (A1) and (P1). *Acinetobacter baumannii* (A1) and *Pseudomonas aeruginosa* (P1) biofilm forming bacteria.

Coated and uncoated silk and vicryl sutures were assessed for their antibiofilm activity against 2 of the most common biofilms forming bacterial isolates, *Acinetobacter baumannii* (A1) and *Pseudomonas aeruginosa* (P1). The results showed that the PVA coated vicryl or silk sutures had the highest optical density, even greater than that of the uncoated silk or vicryl sutures, and the lowest biofilm inhibition among all the tested sutures, Using CS, CS-Ag, PVA-Ag and AgNPs as coating materials on silk and vicryl sutures showed a significant low optical density, indicating the ability of these coating materials to prevent bacterial attachment and cause a high percentage of biofilm inhibition. The most significant biofilm inhibition was observed at 0.3% (w/v) of CS-Ag as a coating material on vicryl sutures causing 89.2 ± 3.1% and 78.3 ± 5.4% inhibition against A1 and P1, respectively. The silk coated sutures showed significant biofilm inhibition of 86.4 ± 1.2% and 72.9 ± 2.3%, against A1 and P1, respectively, at 0.6% (w/v) concentration of AgNPs and nanocomposites as a coating material, as shown in Fig. 13.

### Release of silver ions from coated sutures

The release of Ag from each coated silk and vicryl sutures was calculated after 7 and 14 days. The AAS results showed that the Ag ions released were affected by type of suture and the coating material. As for the silk coated samples, after 7 days they showed the highest amount of Ag ions released with no significant change from that released after 14 days. Amount of Ag ions released ranged from 0.123 to 0.75 ppm for all silk coated sutures and from 0.03 to 0.93 ppm for all vicryl coated suture, along the 14 days. However, the PVA-Ag coated samples, either vicryl or silk sutures showed the highest amount of Ag ions released reaching 0.75 and 0.93 ppm, respectively.

## Discussion

Biogenically synthesized AgNPs were perused in this study. The microorganism, *Enterobacter cloacae* Ism 26 (KP988024), mediates the nucleation and growth of AgNPs^15^. This pathway provides a spherical controlled shape, low aggregation rates, high homogeneity with a low polydispersity index (PI), highly stable nanoparticles and negatively charged nanoparticles that are used in addition to the nanocomposite as a coating layer on absorbable vicryl and nonabsorbable silk sutures. These sutures have been used for wound closure in oral and maxillofacial area such as after tooth extraction, buccal and/or lingual flaps, and flap closure in edentulous ridge. The use of nanoparticles has been reported in other studies ^2,8,18^, which have used various types of sutures to increase the effectiveness of nanomaterials as coating material and their impact on the future of one of the most ancient wound closure devices. In our study, we wanted to compare the same coating materials on different types of sutures; we investigated the efficacy of coating vicryl and silk sutures with AgNPs, CS-Ag or PVA-Ag nanocomposite layer and tested this coating effect on the antimicrobial and antibiofilm activities of these sutures. Silk suture is made of natural protein fiber that is unabsorbable with high affinity to microbial infection, and vicryl suture is synthetic and absorbable type of suture. The impact and effectivity of coating these sutures with AgNPs and nanocomposite layer were detected using various devices. The AFM results confirmed that coating surgical sutures can strongly influence surface topography and cause a significant decrease in surface roughness^19^. This effect was most significantly observed with CS-Ag silk coated sutures. This can be explained by the cationic CS as a highly biocompatible and positively charged polymer, due to the presence of amino groups, with high affinity to negatively charged NPs, enhancing docking on silk suture material. This conclusion was hypothesized by a previous study that used chitosan as a coating material on silk suture that showed a lower coefficient of friction compared to uncoated sutures, and showing uniform coating layer on suture surface^16^. Other study showed higher surface roughness for chitosan coated polyglycolide suture^20,21^ The usage of AFM to study surface topography of sutures before and after coating is very rare in literature and our study results can provide some insight regarding this coated sutures topography^20^. The impact of coating material on surface roughness cannot be ignored, and its impact on antibacterial and antibiofilm activities of CS-Ag coated silk sutures^22^. Upon addition of negatively charged AgNPs to positively charged chitosan polymer, CS acted as the perfect encapsulation form, causing the significant antibacterial activity against Gram positive and Gram negative bacteria^19^. AgNPs and specifically, spherical-shaped nanoparticles have significantly greater antimicrobial effects on various bacterial species^15^. The nanostructured silver has a high surface-to-volume ratio, leading to high efficacy in anchoring to the microorganism’s cell structure and highly penetrating the bacterial cell wall, forming free radicals, damaging DNA, causing structural changes and finally causing bacterial cell death^15^. The release of silver ions from both sutures has impacted the antimicrobial and antibiofilm activity but these effects were augmented upon the addition of CS, which has retained and sustained the coated sutures abilities. AgNPs have broad-spectrum antibacterial activity against gram positive and gram-negative bacteria, and their incorporation into medical devices can lead to increased antimicrobial and antibiofilm activities. These results can explain the extremely high bacterial optical density observed on uncoated silk and vicryl sutures and show that because a barrier can act as an insulator from bacterial attachment, it needs to be augmented with antimicrobial capabilities. This was confirmed by observing the state of PVA-coated sutures which acted as niches for bacterial colonization and did not cause any form of bacterial inhibition; in contrast, they act as attractive agents that amplify infection and colonization. For absorbable vicryl coated sutures, a previous study has shown that CS coated polyglycolide (PGA) a synthetic, absorbable suture, has caused roughness and agglomerated layer that has increased bacterial attachment and colonization^20^. Similar to our results, these findings indicate the direct proportionality between surface roughness and bacterial attachment ability. Here, we can see that the antimicrobial results were complementary and confirmatory to the AFM results. Whereas the surface roughness decreased, bacterial accessibility to the suture surface decreased, and reflected by an increase in the bacterial inhibition zone and a decline in biofilm formation. The increase of AgNPs concentration with vicryl coated was also studied in this paper, where the gradual increase of nanoparticle concentration showed higher antimicrobial and antibiofilm activities till reaching saturation and causing the same inhibition zones. However, as the concentration increased with silk coated sutures, the sutures’ ability to retain nanoparticles and antibacterial sustainability increased. FE-SEM/EDX of the PVA-Ag coated vicryl or silk sutures displayed a thick layer of coating that smoothed the surface of the sutures. This can be explained by adopting the physical adsorption phenomena that includes Van der Waals forces, electrostatic forces, and hydrogen bonding to the ability of negatively charged absorbable polymer with negative oxygen atoms to attach and bond with coating AgNPs and nanocomposites^23^. However, this alteration in surface morphology was not attributed to more AgNPs on the suture surface or higher antimicrobial activity, indicating the possibility of high affinity of PVA for sutures without significant properties, as the presence of this type of coating has led to more bacterial attachment and significantly decreased suture antibiofilm and antibacterial activities. On the contrary, the CS-Ag coated silk sutures exhibited a significant impact on bacterial growth and attachment, leading to significant antimicrobial and antibiofilm activities. This coating material layer acted as a barrier and gave the AgNPs access to the bacterial cells in the surrounding environment to eliminate them and cause the greatest inhibition zones and biofilm inhibition. As a coating layer, AgNPs produced bright spots on the suture surfaces of both the silk and the vicryl sutures, as indicated by the antibacterial activity, which was significantly enhanced upon the addition of CS. Similar results were reported in previous studies^16,18,24,25^. The accessibility of AgNPs is highly influenced by their scaffold. Compared with nanoparticles alone, the ability of CS and PVA to carry AgNPs increased the inhibition zone and antibiofilm effectiveness. The coating layer has helped in acting as a slippery layer that prevented bacterial proliferation and inhibited biofilm formation^26^. This can be explained by the ability of cationic polymer such as chitosan to provide a high positive charge to the NPs, enhancing docking and encapsulation. On the other hand, nonionic polymer like PVA can affect NPs attachment and cause loss of silver nanoparticles and impact the antimicrobial and antibiofilm activities^27^. Furthermore, the antimicrobial activity of CS alone cannot be forgotten where the positively charged amino groups are the main factor behind the interaction between CS and negatively charged bacterial surface leading to bacterial cell leakage and eventually bacterial death^28^. This effect been multiplied by the addition of AgNPs, resulting in the highest rates of bacterial and biofilm inhibition^4,29,30^. Therefore, augmenting nanoparticles is an essential step in our research, as implementing lower concentrations of nanomaterials in addition to an effective carrier can channel these particles to their designated target. The Addition of this thin layer of coating on the suture surface has positively impacted the fight against MDR bacteria and SSI. Similar results were recorded in previous studies^6,31,32^. The presence of encapsulated nanomaterial and nanoparticles that function as antimicrobial agents, effectively attached to surgical devices, has significantly improved the ability of these devices to resist bacterial attachment and can be used as a superior choice to overcome current multidrug-resistant bacterial infections that infect foreign medical devices^1,9,13^. AgNPs can release Ag ions from silver clusters, and this continuous release ensures the durability of their antimicrobial activity. They are highly accessible to bacterial cells because of their nanosized range; therefore, their antimicrobial activity is uniquely high^9,15^. The literature lacks comparative studies analyzing the same coating materials against different types of sutures^33^. In our study we have compared silk coated sutures with vicryl coated ones using the same coating materials. This study has investigated the impact of surface topography, different coatings and different suture types on bacterial colonization in comparison to uncoated sutures. Further studies comparing coating materials against different types of suture are needed to reach conclusive data regarding the same coating layers and future in vivo studies are inevitable for better understanding of the coated sutures ability to minimize suture site infections and help in our fight against the non-stop emergence of multi drug resistant bacteria^33,34^.

## Electronic supplementary material

Below is the link to the electronic supplementary material.

Supplementary Material 1

## Data Availability

The data are available from the corresponding authors upon reasonable request.
