# Supplementary material for: An in vitro study on the efficacy of nanoparticles and nanocomposites as coating materials on surgical sutures
Source: Sci Rep. 2025 Jul 1;15:20368. doi: 10.1038/s41598-025-07558-6 (PMC12218398; doi:10.1038/s41598-025-07558-6)
Supplement: Supplementary file 1 — Supplementary Material 1 [file 41598_2025_7558_MOESM1_ESM.pdf]

Supplementary materials :

Vicryl uncoated and coated raw AFM data

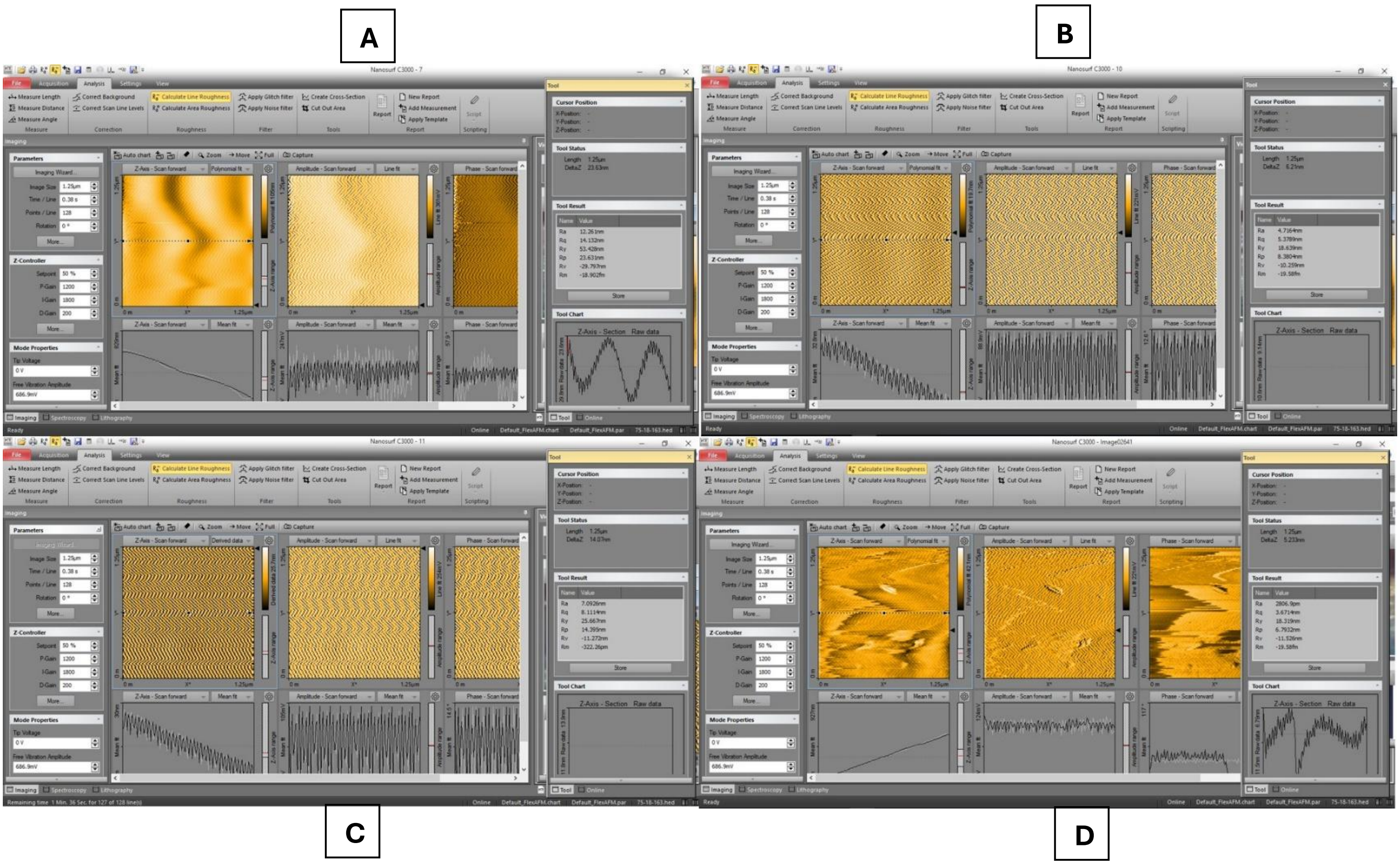

Supplementary Fig S1: AFM results for uncoated and coated Vicryl sutures. A) uncoated vicryl suture, B) CS-Ag coated vicryl suture, C) PVA-Ag coated vicryl suture and D) AgNPs vicryl coated sutures

Silk uncoated and coated raw AM data

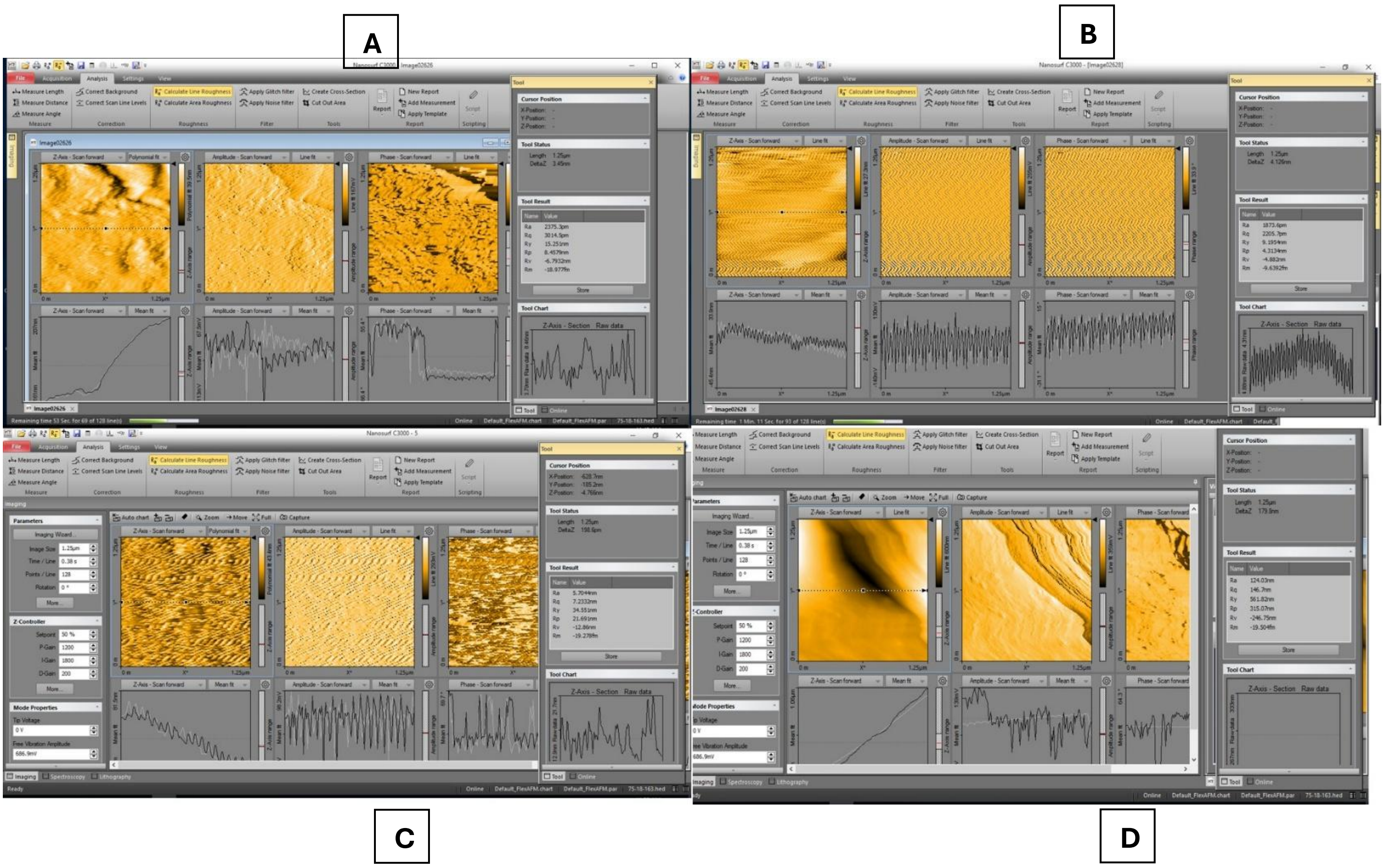

Supplementary Fig S2: AFM results for uncoated and coated Silk sutures. A) uncoated Silk suture, B) CS-Ag coated Silk suture, C) PVA-Ag coated Silk suture and D) AgNPs Silk coated sutures
